# Disseminating FBT to a private practice setting: practicalities and pitfalls

**DOI:** 10.1186/2050-2974-3-S1-O40

**Published:** 2015-11-23

**Authors:** Mandy Goldstein, Christopher E Thornton

**Affiliations:** 1 The Redleaf Practice, Australia

## 

The use of effective treatments for eating disorders has been emphasised, given their well-known risks. Despite this, the research-practice gap persists. The Redleaf Practice recently undertook an evaluation to explore the effectiveness of family-based treatment (FBT) for adolescent anorexia nervosa in a private practice setting. The study, while supporting the use of FBT in private practice, raised questions about barriers to the uptake of this treatment in this setting. This paper will therefore present an exploration of these challenges, and will offer practical recommendations to those considering the use of FBT in alternate treatment settings.

